# Layered Oxygen-Deficient Double Perovskites as Promising Cathode Materials for Solid Oxide Fuel Cells

**DOI:** 10.3390/ma15010141

**Published:** 2021-12-25

**Authors:** Andrei I. Klyndyuk, Ekaterina A. Chizhova, Dzmitry S. Kharytonau, Dmitry A. Medvedev

**Affiliations:** 1 Department of Physical, Colloid and Analytical Chemistry, Organic Substances Technology Faculty, Belarusian State Technological University, Sverdlova 13a, 220006 Minsk, Belarus; chizhova@belstu.by; 2 Jerzy Haber Institute of Catalysis and Surface Chemistry, Polish Academy of Sciences, Niezapominajek 8, 30-239 Krakow, Poland; dmitry.kharitonov@ikifp.edu.pl; 3 Laboratory of Electrochemical Devices Based on Solid Oxide Proton Electrolytes, Institute of High Temperature Electrochemistry, Ural Branch of Russian Academy of Sciences, 620660 Ekaterinburg, Russia; dmitrymedv@mail.ru; 4 Hydrogen Energy Laboratory, Ural Federal University, 620002 Ekaterinburg, Russia

**Keywords:** SOFCs, SOECs, layered perovskite, double perovskite, cobaltites, oxygen deficiency, cathode materials, thermal expansion, thermal stability, electrical transport, electrochemistry, energy conversion

## Abstract

Development of new functional materials with improved characteristics for solid oxide fuel cells (SOFCs) and solid oxide electrolysis cells (SOECs) is one of the most important tasks of modern materials science. High electrocatalytic activity in oxygen reduction reactions (ORR), chemical and thermomechanical compatibility with solid electrolytes, as well as stability at elevated temperatures are the most important requirements for cathode materials utilized in SOFCs. Layered oxygen-deficient double perovskites possess the complex of the above-mentioned properties, being one of the most promising cathode materials operating at intermediate temperatures. The present review summarizes the data available in the literature concerning crystal structure, thermal, electrotransport-related, and other functional properties (including electrochemical performance in ORR) of these materials. The main emphasis is placed on the state-of-art approaches to improving the functional characteristics of these complex oxides.

## 1. Introduction

Fuel cells (FCs) are electrochemical devices in which the chemical energies of different fuels (including fossil fuels) can be directly and effectively converted into electrical energy in one stage [1,2]. In FCs the restrictions of the Carnot cycle are absent; therefore, the thermodynamic efficiency of these devices may reach 90% and higher [3] depending on their composition, interval of working temperatures, etc. These devices are categorized into different groups which consider geometrical design [1], fuel (hydrogen, biomass, hydrocarbons, alcohols, etc.) [2], used electrolyte (proton-conducting membranes, oxygen- and proton-conducting solid oxide fuel cells (SOFCs), etc.) [3], and operating temperature range (low-temperature, intermediate-temperature, and high-temperature FCs), etc. Taking into account peculiarities of the SOFC’s design, they can be divided into typical asymmetrical (A–SOFCs, in which the cathode and the anode are made of different materials) and symmetrical (S–SOFCs, in which the cathode and the anode materials are the same) derivatives [4]. Typically, SOFCs are divided into three groups according to their operation temperatures (and the solid electrolyte used in them): high-temperature SOFCs (HT–SOFCs, 1073–1273 K, and ZrO_2_- or CeO_2_-based solid electrolytes), intermediate–temperature SOFCs (IT–SOFCs, 873–1073 K, and LaGaO_3_-based solid electrolytes), and low–temperature SOFCs (LT–SOFCs, < 873 K, and δ-Bi_2_O_3_ or Bi_4_V_2_O_11_-based solid electrolytes) [5,6,7,8,9,10]. Usually, oxygen-ion-conducting solid electrolytes are widely used in SOFCs (such as the above mentioned electrolytes and others [5,6,7,8,9,10]), but recently increasing attention has been paid to proton-conducting BaCeO_3_- and BaZrO_3_-based solid electrolytes [2,8,10,11], which exhibit some advantages over oxygen-ionic electrolytes, especially at intermediate and low temperatures.

Despite essential progress, which recently has been achieved in the development of individual SOFCs materials (electrolytes, anodes, cathodes, collectors, and sealants) [3,6,7,8,9,10,12,13,14,15,16,17,18,19,20] and the production and testing of SOFCs [1,2,7,21,22,23], some urgent challenges still exist. One of the most important of these challenges is the search for, development of, and study of new electrode materials, which possess high stability, thermal and chemical compatibility, and improved electrochemical performance towards cathode and electrode reactions occurring in SOFCs. Regarding anodes (fuel electrodes), this problem was partially solved by the development of a new class of anode materials based on double perovskite molybdates SrMMoO_6_ (M = Ni, Mg, and Fe) and their derivatives, including cermets [4,24,25]. Discussing the cathodes (oxygen or air electrodes), one of the most interesting and promising materials for IT–SOFCs are the layered oxygen-deficient double perovskites, *Ln*Ba*M*_2_O_6–δ_ (*Ln*—rare-earth element; *M—*3*d*-metal), which have been intensively studied with this aim for last two decades [4,8,9,10]. Due to the high tolerance of perovskite structure, these complex oxides can be formed at different combinations of their constituents, demonstrating outstanding thermal, electrotransport-related, magnetic, electrochemical, and other properties. As a result, these double perovskites can be used as working elements of chemical gas sensors, high-temperature thermoelectrics, cathode materials of IT–SOFCs, etc. [26].

The present article provides an overview of peculiarities of the crystal structure, physicochemical properties, and electrochemical performance of layered oxygen-deficient perovskites as cathode materials of SOFCs, as well as ways and methods of improving the functional characteristics of these materials.

## 2. Cathode Materials for IT–SOFCs: Past, Present, and Future

An ideal cathode material for a SOFC must possess [27,28,29,30,31,32,33]: (a) high electronic (*n*- or *p*-type) conductivity (in an oxidizing atmosphere, preferably, more than 10^2^ S cm^−1^); (b) thermal and chemical compatibility with solid electrolytes and interconnectors; (c) enough large porosity to provide fast diffusion of gaseous oxygen through a cathode to a triple-phase cathode–solid electrolyte–gas phase interface; (d) high stability in an oxidizing atmosphere; (e) high catalytic activity in an oxygen reduction reaction (ORR); and (f) low cost.

**Figure 1:**
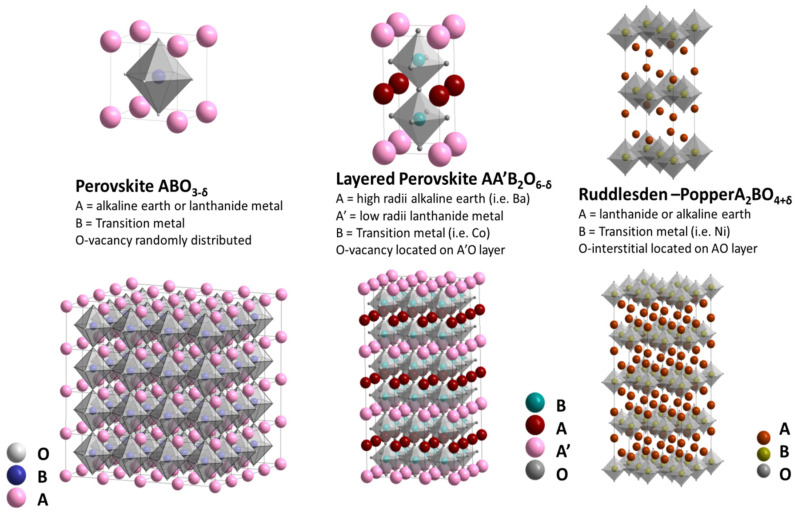
Crystal structures of perovskite, double perovskite, and Ruddlesden–Popper phase (Reproduced from [40] with permission from the Royal Society of Chemistry).

Typical cathode materials utilized in SOFCs are perovskites (ABO_3_) of light rare-earth elements (REE), 3*d*-metals (usually, Mn, Fe, Co, and Ni), and their solid solutions and composites [4,6,7,8,9,10,27,34,35,36,37,38,39]. Among all cathode materials, Co-based perovskites and their derivatives received enormous research interest and played an integral role in the development and commercialization of IT–SOFCs [14,21,26,39]. Figure 1 shows the crystal structures of perovskites and other complex oxides possessing mixed ionic–electronic conductivity (MIEC), which have been used as cathode materials for SOFCs operating in different temperature intervals. A common strategy for tuning their functional properties is the partial heterovalent substitution of A- or/and B-site cations in their structure, mostly to enhance electrical conductivity and to lower the value of the thermal expansion coefficient (TEC). To decrease the electrode polarization losses at reduced operating temperatures, the addition of noble metals (Pd, Ag, and Pt) to the cathode material is used as well [27]. To increase the compatibility of these compounds with the solid electrolyte, they are often used in the form of composites.

During the investigation of ABO_3_ perovskite solid solutions, a new class of oxide materials (named layered oxygen-deficient double perovskites (LODPs)), *Ln*Ba(*M*’,*M*”)_2_O_6–δ_ (*Ln* is REE; *M*’, *M*” are 3*d*-metals) (cation-ordered phases), was purposefully designed. Their electrochemical performance is reported to be better than that of the parent perovskites. Therefore, these phases are currently considered as being very promising cathode materials, especially for IT–SOFCs [4,6,8,9,10,34,35,40,41] and have been intensively studied both as single materials and SOFCs components [42].

Another group of cathode materials includes complex oxides with so-called Ruddlesden–Popper (RP) phases (layered oxides) with a general formula of A*_n_*_+1_B*_n_*O_3*n*+1_. These compounds, for example, *Ln*_2_NiO_4+δ_ (*Ln* = La, Pr, Nd), and their solid solutions possess high diffusivity of interstitial oxygen ions, comparatively low TEC values, and high enough electrical conductivity, which also makes them promising for SOFCs applications [6,8,10,27,40]. Other RP phases, such as (Sr,La)_3_(Fe,Co)_2_O_7–*x*_ (*n* = 2) and (La,Sr)_4_(Fe,Co)_3_O_10−*x*_ (*n* = 3) showed good electrocatalytic activity in ORR in single cell measurements [6].

Recently, it has been shown that effective cathodes for proton-conducting SOFCs operating at low and intermediate temperatures are Ba(Ce,Zr)O_3_-based solid solutions doped with transition elements in high concentration. These phases demonstrate excellent chemical compatibility with typical proton-conducting solid electrolytes due to the similarity of their compositions [43,44]. These compounds belong to the triple-conducting oxides (TCOs) [45,46,47], in which transport species are simultaneously protons, oxygen ions, and electrons (holes). The advantage of TCOs as cathode materials for proton-conducting SOFCs is that both protons from the solid electrolyte and oxygen species adsorbed from the air may migrate through the bulk and over the surface of the cathode, which extends the reaction area over the whole electrode. We should note that LODPs, such as *Ln*Ba_0.5_Sr_0.5_Co_1.5_Fe_0.5_O_5+δ_ (*Ln* = Pr, Nd), when used as cathodes for proton-conducting SOFCs, also belong to the class of TCOs [9,44]. According to [48], triple conductivity in LODPs can appear due to the formation of impurity phases, which should be taken into account for the development of new TCOs.

**Figure 2:**
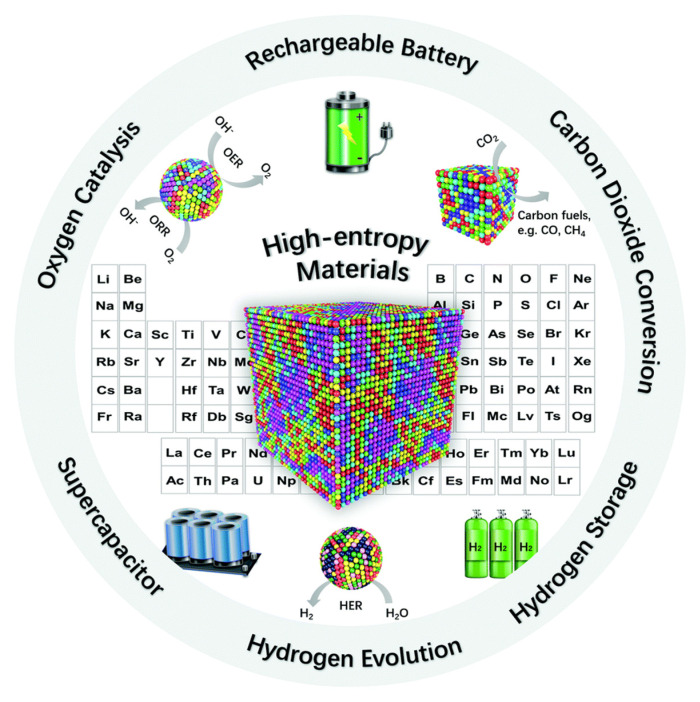
Fields of using high-entropy materials for energy conversion and storage purposes (Reproduced from [49] with permission from the Royal Society of Chemistry).

Very interesting and promising materials for use in SOFCs are high-entropy ceramics (HECs, Figure 2), which are solid solutions of inorganic compounds (including metal oxides) with one or more Wyckoff site shared by equal or near-equal atomic ratios of five (or occasionally four) constituting elements [49,50]. The so-called compositionally complex ceramics (CCCs) include, besides HECs, medium-entropy and/or non-equimolar compositions [51]. It has been shown recently that medium- and high-entropy perovskite oxides, such as Sr(Fe_α_Ti_β_Co_γ_Mn_ζ_)O_3–δ_ [52], (La,Sr)(Co,Cr,Fe,Mn,Ni)O_3–δ_ [53,54], and (La,Pr,Nd,Sm,Ba,Sr)(Co,Fe,Ni,Cu)O_3–δ_ [55] demonstrate lower TECs with a lack of visible contribution from the chemical expansion effect and a more stable and much lower polarization resistance compared with the conventional cathode materials. These peculiarities make such materials very attractive for electrochemical applications.

## 3. Crystal Structure, Phase Transitions, and Physicochemical and Functional Properties of Layered Oxygen-Deficient Double Perovskites

The crystal structure of *Ln*Ba(*M*’,*M*”)_2_O_6–δ_ LODPs, consisting of alternating (–BaO–), (–(*M*’,*M*”)O_2_–) and *Ln*O_1–δ_ layers, is formed due to the ordering of oxygen vacancies, *Ln*, and Ba atoms in the structure of oxygen deficient *LnM*’(*M*”)O_3–δ_ perovskites according to Equation (1):

2*Ln*_0.5_Ba_0.5_CoO_3–δ_ (*Pm*3*m*) →*Ln*BaCo_2_O_6–δ_ (*P*4/*mmm* or *Pmmm*) (1)

**Figure 3:**
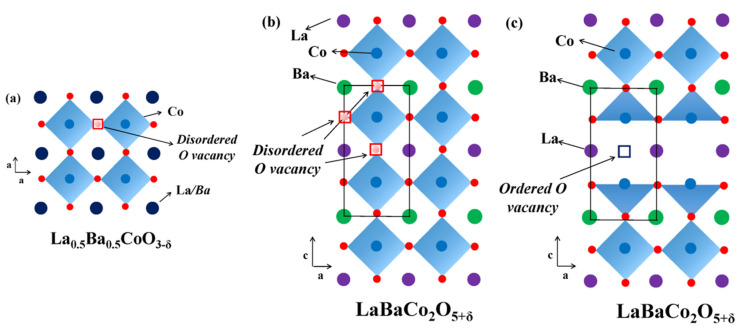
Crystal structures of (**a**) cubic *Pm*3*m* single perovskite La_0.5_Ba_0.5_CoO_3–δ_; (**b**) tetragonal *P*4/*mmm* ordered A-site cation and disordered oxygen-vacancy double perovskite LaBaCo_2_O_5+δ_; (**c**) orthorhombic *Pmmm* both an ordered A-site cation and ordered oxygen-vacancy double perovskite LaBaCo_2_O_5+δ_ (reproduced with permission from [56]. Copyright 2016, Multidisciplinary Digital Publishing Institute).

A structural phase transition occurs at such an arrangement: cubic perovskite structure (*Pm*3*m*) transforms into a tetragonal (*P*4/*mmm*) or an orthorhombic (*Pmmm*) structure (Figure 3). A different character of oxygen-vacancy ordering, as well as the ordering of *M*’ and *M*” ions, may result in the fact that LODPs crystallize in other space groups (SGs), such as *P*1, *P*2, or *Ammm* [26]; nevertheless, *P*4/*mmm* or *Pmmm* are the most typical ones.

This transition usually occurs at high temperatures and low oxygen partial pressures and may proceed through the formation of an intermediate product with a complex domain structure, which has a strong affinity with oxygen and may exchange it with an atmosphere at relatively low temperatures (ca. 340 K) [57].

Oxygen content variation in LODPs results in a change in their structure, which takes place at different temperatures depending on the cationic composition and oxygen partial pressure. For example, GdBaCo_2–*x*_Fe*_x_*O_6–δ_ (0 ≤ *x* ≤ 0.4) perovskites undergo a *Pmmm*–*P*4/*mmm* phase transition at approximately 730–760 K [58]. Layered cobaltites of REE and barium undergo the dielectric-metal transition, in which their electrical conductivity increases by several orders of magnitude. This is caused by a change in the spin state of the cobalt ions [26,59]. At high temperatures, these phases are paramagnetic, but at cooling LODPs become antiferro-, ferri-, or ferromagnetic [26,60,61,62] due to a different type of ordering of magnetic moments of the transition metal ions in their structure.

Electrical conductivity values of LODPs vary widely depending on their cationic composition and oxygen nonstoichiometry [26,58,60,62,63,64,65,66,67]. For compounds which contain light REEs and possess small oxygen deficiencies, electrical conductivity is approximately 10^3^ S cm^−1^ (such phases are metal-like conductors). On the contrary, for compounds composed of heavy REE and possessing large oxygen deficiency, conductivity is approximately 10^−8^ S cm^−1^, which is typical for dielectrics.

Large values of both the Seebeck coefficient and the electrical conductivity of several layered cobaltites [58,68], ferrocobaltites [62,69], ferrocuprates [69,70,71,72], and other LODPs [73] make them promising candidates for use in high-temperature thermoelectrogenerators (TEGs) for effective conversion of heat into electrical energy.

Double perovskites *Ln*BaCo_2_O_5+δ_ (*Ln* = Eu, Gd, and Sm) containing weakly-bonded oxygen (δ) and cobalt ions in different oxidation states (Co^2+^, Co^3+^, Co^4+^) demonstrate certain photocatalytic activity during the degradation of Congo Red, which indicates that they may be promising photocatalysts for the oxidation (degradation) of organic substances [74].

To be used in high-temperature devices, such as SOFCs, solid oxide electrolysis cells (SOECs), TEGs, and other applied directions, LODPs must possess a good thermomechanical and chemical compatibility with other components of these devices [75]. The first condition of thermomechanical compatibility is the similarity of their TEC values with those of the typical solid electrolytes used in SOFCs. The TEC values for some typical LODPs are set out in Table 1. As can be seen, for the layered perovskites with only Co atoms in B-site, the TEC values vary within ca. (17−24) × 10^−6^ K^−1^; the measured TECs are much higher than those of the commonly used ZrO_2_-, CeO_2_-, and LaGaO_3_-based solid electrolytes (as a rule, between (10−13) × 10^−6^K^−1^) [5].

The TEC values of layered oxygen-deficient double cobaltites decline when the REEs’ ionic radii decrease, as well as when a partial substitution of cobalt with iron (at small doping levels) or copper occurs (Table 1). Both of these strategies are effective in improving the thermomechanical properties of LODPs. Molecular dynamics simulations have shown [76], that variations in the cationic composition or/and oxygen nonstoichiometry of layered oxygen-deficient double cobaltites is the other effective way to reduce the TEC values of these materials.

The TEC values of LODPs sharply increase at high temperatures (above ca. 500–700 K) due to the beginning of the evolution of weakly-bonded oxygen from their crystal structures into the environment. Therefore, the expansion of these phases at high temperatures is determined by both thermal and chemical factors [26,58,62,75].

The chemical expansion coefficient (CEC) values of some LODPs are provided in Table 2. As can be seen, these values vary within 0.5–2.6%, exhibiting a strong anisotropy; simultaneous chemical expansion along the *a*-axis (in the *ab*-plane) and chemical contraction along the *c*-axis (out-of-plane) take place during the evolution of weakly-bonded oxygen. According to [58,77], the former occurs due to an increase inthe average radii of cobalt ions due to their reduction, but the latter occurs due to the change in the coordination environment of REE and cobalt ions.

A serious drawback of layered cobaltites is their degradation in CO_2_-containing atmospheres. For example, Zhu et al. [81] have studied the degradation features of PrBaCo_2_O_5+δ_in CO_2_-containing atmospheres. They found a considerable decrease in the electrochemical activity of PrBaCo_2_O_5+δ_ electrodes due to the formation of insulating BaCO_3_ particles at the PrBaCo_2_O_5+δ_ surface.

On the other side, efficient cathodes of SOFCs should provide a large oxygen exchange rate between the atmosphere and the surface of the cathode and enough high oxygen mobility. Therefore, both bulk and surface chemistry in the oxygen exchange kinetics of MIEC layered perovskites are very important. It was found [82] that the surfaces of PrBaCo_2_O_5+δ_ and GdBaCo_2_O_5+δ_ LODPs can significantly change in their local chemical composition and can exchange by oxygen with the atmosphere even at ambient temperature.

Perry and Ishihara [83] summarized the main directions of improving the efficiency and durability of oxygen electrodes (including those based on LODPs) in SOFCs. Concerning the bulk chemistry, the areas of “electro-chemo-mechanics” are (1) the enhancement in transport and surface reactivity through the strain-state tailoring (mechano-electrical and mechano-electrochemical coupling) and (2) the mitigation of deleterious chemical expansion during operation, induced by stoichiometry changes (chemo-mechanical coupling). Regarding the surface chemistry and oxygen surface exchange kinetics, the main areas of interest are (1) clarifying the rate-limiting steps and mechanisms of oxygen incorporation/excorporation with atomistic insight; (2) exploiting the unique properties of hetero-interfaces, grain boundaries, and other large surface defects; (3) identifying the optimal composition for the outermost atomic monolayers; and (4) studying how to control the outermost chemistry in operating conditions via bulk and surface chemical tailoring.

Oxygen mobility in materials used in SOFCs and catalytic membranes was comparatively discussed in detail in [84]. The results of the investigation of chemical compatibility between different oxide electrodes and solid electrolytes in SOFCs are provided in [85]. Concerning LODPs, cathode materials such as *Ln*BaCo_2_O_5+δ_ (*Ln* = Pr and Gd) possess poor chemical compatibility with (Ce,Sm)O_2–δ_ (SDC) and(Ce,Gd)O_2–δ_ (GDC) above 1173 K and are prone to chemical reactions with the formations of BaCeO_3_, BaCoO_3_, and Sm_2_CuO_4_. The composite material of the LODP cathode with (La,Sr)(Cr,Mn)O_3_ (LSCM) is stable below 1273 K without any obvious secondary phase formation. However, chemical interactions in this material may occur above 1473 K. Chemical reactions between the LODP cathode and Ba(Zr,Y)O_3–δ_ (BZY) or Ba(Zr,Y,Yb)O_3–δ_ (BZYYb) proton conductors were not observed. However, GdBaCo_2_O_5+δ_ reacts with YSZ at 973 K with the formation of BaZrO_3_. Doping with Fe, Cu, and Nb at the B-site of *R*BaCo_2_O_5+δ_ (*R* = Pr and Y) can effectively improve chemical compatibility between the LODP cathodes and SDC or GDC solid electrolytes.

**Figure 4:**
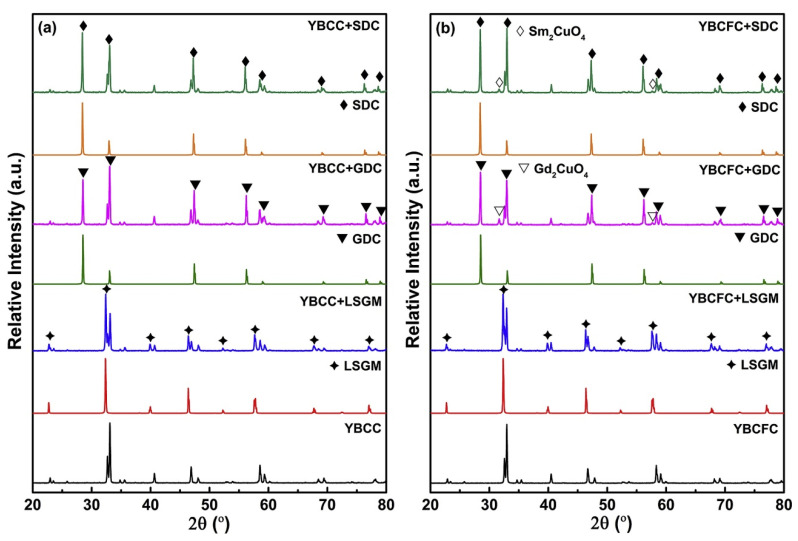
XRD patterns of the samples: YBCCO–SDC, YBCCO –GDC, and YBCCO –LSCM: (**a**) YBCFC–SDC, YBCFC–GDC, and YBCFC–LSCM (**b**) calcined at 1123 K for 10 h in air (reproduced with permission from [86]. Copyright 2019, Elsevier).

Figure 4 shows the XRD results for YBaCoCuO_5+δ_ (YBCC) and YBaCo_2/3_Fe_2/3_Cu_2/3_O_5+δ_ (YBCFC), which were calcined at 1223 K for 10 h in air mixtures with different solid electrolytes (SDC, GDC, and LSCM). No impurities or shifts of diffraction peaks were observed for compositions containing YBCC (Figure 4a), indicating that this compound is chemically compatible with the studied electrolytes. On the contrary, small amounts of Sm_2_CuO_4_ and Gd_2_CuO_4_ impurity phases in the YBCFC–SDC and YBCFC–GDC mixtures were observed (Figure 4b), showing that the YBCFC material is incompatible with SDC and GDC electrolytes. However, the absence of impurity phases in the YBCFC–LSCM mixture indicates that YBCFC presents good chemical compatibility with LSCM solid electrolytes below 1223 K.

Tsvetkov et al. [87] found that PrBaCo_2_O_5+δ_ and SDC experienced the interdiffusion of Pr and Sm at 1273 K; the formation of BaCeO_3_ had high electrical resistivity. Diffusion of praseodymium into the electrolyte resulted in an increase in its electronic conductivity, which led to adecrease in the open-circuit voltage and even the short circuit of the cell [88]. The formation of the BaCeO_3_ phase also increased the ohmic and polarization resistance of the components of the electrochemical cell.

The NdBaFe_1.9_Mn_0.1_O_5+δ_ cathode material showed good chemical compatibility with the BaZr_0.1_Ce_0.7_Y_0.2_O_3–δ_ (BZCY) electrolyte at a temperature of 1173 K [89]. In [90], it was shown that Sr_2_(Co,Nb)FeO_5+δ_ double perovskites do not react with La_0.9_Sr_0.1_Ga_0.8_Mg_0.2_O_3_ (LSGM) at 1273 K in air, indicating the good chemical compatibility of these compounds.

## 4. Electrochemical Performance of Layered Oxygen-Deficient Perovskites

### 4.1. Layered Cobaltites of REEs and Barium

The main advantages of layered oxygen-deficient cobaltites as cathodes for SOFCs are their superior electronic and ionic conductivity, as well as their higher electrocatalytic activity towards the ORR, especially in the IT-temperature range [8,9,10,34,35,40]. Table 3 summarizes the electrochemical performance of REE–barium layered cobaltites.

According to this table, layered cobaltites exhibit good electrochemical activity with the lowest ASR value of 0.0086 Ω cm^2^ at 1073 K reached for the LaBaCo_2_O_5+δ_ cathode. This is much lower than values taken for commonly used LODPs. The ASR value for the NdBaCo_2_O_5+δ_ cathode is 0.08 Ω cm^2^ at 973 K, under a cathodic applied voltage of −0.1 V [96]. The activation energy of the interface conductivity of these materials varied within ~110 kJ mol^–1^ and 160 kJ mol^–1^, decreasing with an increase in the applied voltage (*E*), since the diffusion process was more easily affected by the increase in *E* than the charge transfer process.

**Figure 5:**
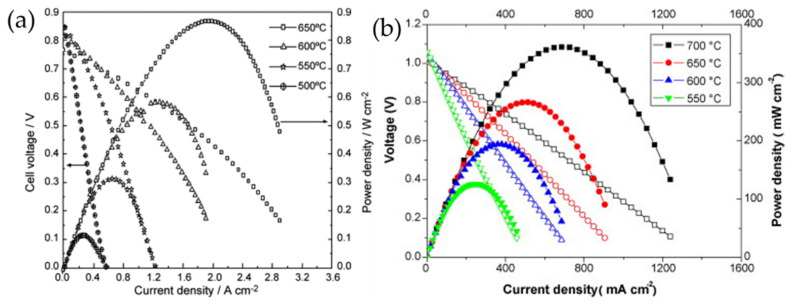
Performances of the cell with PrBaCo_2_O_5+δ_ cathode and (**a**) oxygen-ion-conducting Ce_0.8_Sm_0.2_O_1.9_ electrolyte (reproduced with permission from [69]. Copyright 2008, Elsevier) and (**b**) proton-conducting BaCe_0.5_Zr_0.3_Y_0.16_Zn_0.04_O_3–δ_ electrolyte (reproduced with permission from [98]. Copyright 2010, Elsevier).

The highest power density values were obtained for PrBaCo_2_O_5+δ_, which reached 866 mW cm^−2^ at 923 K for the cell (−)NiO|Ce_0.8_Sm_0.2_O_1.9_|PrBaCo_2_O_5+δ_(+) and 361 mWcm^−2^ at 973 K for the cell (−)NiO+BaCe_0.5_Zr_0.3_Y_0.16_Zn_0.04_O_3–δ_|BaCe_0.5_Zr_0.3_Y_0.16_Zn_0.04_O_3–δ_|PrBaCo_2_O_5+δ_(+) (Figure 5). It is interesting to note that the electrochemical performance of this material in SOFCs containing oxygen-ion conducting solid electrolytes is essentially higher than that in SOFCs with the proton-conducting electrolyte.

As a whole, the overall electrochemical performance of the ordered oxygen-deficient double perovskites is reported to be higher than for A-site disordered phases. However, the performance of *Ln*BaCo_2_O_5+δ_ cathodes usually deteriorates with decreases in the *Ln*^3+^ ionic radius, partly due to a decrease in oxygen content [94,99,100,101]. The reported ASR values for the La_0.5_Ba_0.5_CoO_3–δ_ and LaBaCo_2_O_5+δ_ cathodes at 873 K were equal to 11.5 and 7.4 Ω cm^2^, respectively, with activation energy values of 0.90 and 0.97 eV, respectively [102].

The electrocatalytic activity and stability of *Ln*BaCo_2_O_5+δ_ (*Ln* = La, Pr, Nd, Sm, Eu, and Gd) perovskites in the hydrogen evolution reaction (HER) were studied in [103]. It was found, from the DFT calculations, that *Ln*BaCo_2_O_5+δ_ phases exhibit an optimal free energy combination for the H_2_O adsorption/dissociation and –OH/H* desorption, which open up the opportunity for the development of new perovskite-based energy materials.

### 4.2. A-Site-Deficient and A-Site-Substituted LnBaCo_2_O_5+δ_ Layered Perovskites

An effective way to improve functional properties of the discussed layered perovskites is through the creation of a cation deficiency in their A-sublattice (both in REE and barium positions) and partial isovalent substitution of barium with smaller alkaline-earth elements (AEEs), such as strontium or calcium. Table 4 summarizes some results obtained for SOFCs based on such double perovskites.

Single-phase materials are formed at a relatively small deficiency of REE (8 mol.% for Pr [104], 5–10 mol.% for Sm [105,106]). The formation of REE-vacancies in the *Ln*_1−*x*_BaCo_2_O_5+δ_ phases leads to an increase in their lattice constants and decreases their oxygen content. This results in a decrease of their electrical conductivity, and improvement of their electrochemical performance (particularly, to the essential lowering of ASR and increasing of PD [105,106,107]), at least at a low REE deficiency level (approximately5 mol.%).

The crystal structure of *Ln*Ba_1–*x*_Co_2_O_5+δ_ is retained at *x* ≤ 0.15, 0.08–0.10 and 0.05 for *Ln* = La [108], Pr [109,110], and Nd [111], respectively. The formation of a barium deficiency slightly affects the lattice constants of LODPs [111] but results in a decrease in the oxygen content [111], their TECs [109,110], and electrical conductivity [108,109,110,111].

A paramount electrochemical performance of 1.03 W cm^−2^ at 973 K was observed in the anode-supported NiO–Ce_0.9_Gd_0.1_O_1.95_|Ce_0.9_Gd_0.1_O_1.95_|PrBa_0.94_Co_2_O_5+δ_ SOFC [110]. Improvement of functional properties was also observed for Ba-deficient solid solutions, such as Pr_0.5_Ba_0.25–*x*_Ca_0.25_CoO_3–δ_ [112] and PrBa_0.5–*x*_Sr_0.5_Co_2_O_5+δ_ [113].

The addition of potassium results in a higher cation deficiency in the PrBa_1−*x*_Co_2_O_5+δ_ perovskites, improving bulk oxygen transport [114]. However, the cobalt content at the surface of these samples was found to have decreased as well, causing the deterioration of their electrochemical performance towards the surface oxygen exchange.

Inter-substitution of praseodymium by barium in Pr_1+*x*_Ba_1−*x*_Co_2_O_6–δ_ leads to the formation of double-phase composites comprising orthorhombic PrBaCo_2_O_6–δ_ (SG *Pmmm*) and PrCoO_3_ (SG *Pnma*) for *x* = 0.2 and 0.8 [115]. The triple-phase boundary reaction suggests the formation of Co(OH)_3_ along with the H_2_ gas evolution during the electrochemical dissolution of these composite electrodes with H_2_O inreaction (2).

Pr_1+*x*_Ba_1−*x*_Co_2_O_6–δ_ + 3H_2_O → Pr_1+*x*_Ba_1−*x*_Co_2−*y*_O_6–δ_ + *y*Co(OH)_3_ + {(6 − 3*y*)/2}H_2_↑ + (3 − 3*y*− δ)/2}O_2_↑ (2)

The sample with *x* = 0.6 showed a higher ORR rate with more intense H_2_ gas evolution compared with the others.

**Figure 6:**
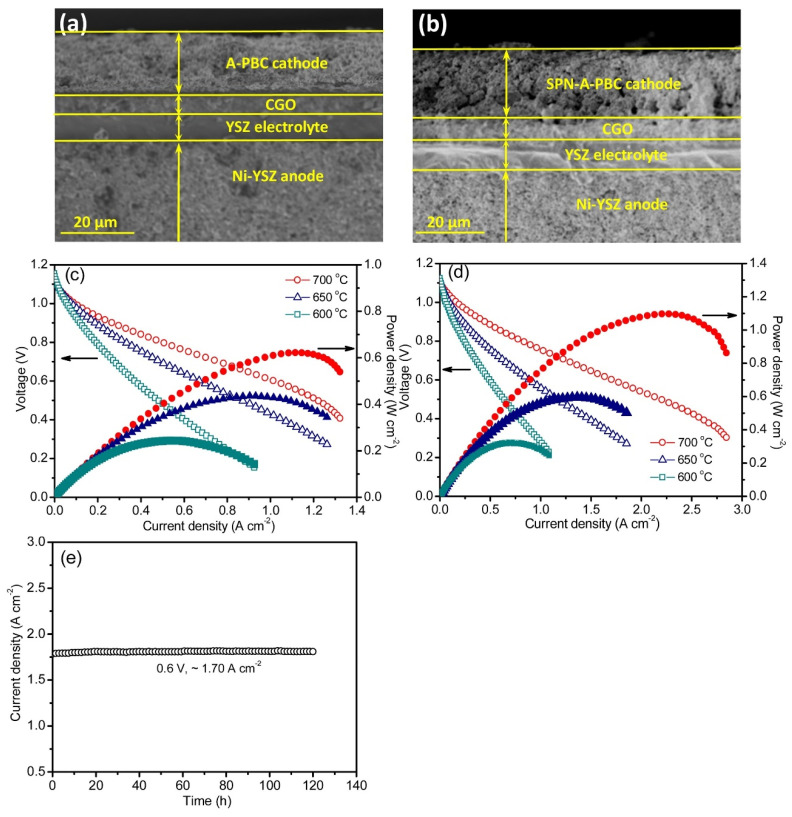
Cross-section view of SEM images of the anode-supported single cell with a Ni–YSZ anode, an YSZ electrolyte, a CGO buffer layer and the (**a**) A–PBC and (**b**) SPN–A–PBC cathodes. *V*–*I* and *P*–*I* curves of the single cells with the (**c**) A–PBC and (**d**) SPN–A–PBC cathodes within 873–973 K. (**e**) Long-term stability test of a single cell with the SPN–A–PBC cathode at a constant loading voltage of 0.6 V at 973 K for 120 h (reproduced with permission from [116]. Copyright 2019, Elsevier).

Lu et al. [116], using the conventional solid-state reactions method, synthesized an A-site deficient double perovskite PrBa_0.94_Co_2_O_5+δ_ (A–PBC) and then created nanorods of simple perovskite (PrCoO_3_) on the surface of its particles via an in situ exsolution process, which resulted in the formation of a heterostructured simple perovskite nanorod-decorated double perovskite cathode (SPN–A–PBC). High electrocatalytic activity of the SPN-A-PBC cathode toward ORR was found, achieving apolarization resistance of about 0.025 Ω cm^2^ at 973 K in air. The anode-supported single cell with the SPN–A–PBC cathode reached a power density of 1.1 W cm^−2^ at 973 K and a superior steady operation over 120 h at a loading voltage of 0.6 V (Figure 6). This electrode also exhibited a good tolerance to CO_2_; when tested in air with 6 vol.% CO_2_ at 973K, it maintained a stable polarization resistance of about 0.078 Ω cm^2^.

A partial substitution of barium with smaller strontium or/and calcium in *Ln*BaCo_2_O_5+δ_ leads to the expected decrease of lattice constants and TEC values [117,118,119,120,124] and an increase in the electrical conductivity [118,123,124] and the electrochemical performance of the corresponding materials [117,118,119,120,121,122,123,124].

**Figure 7:**
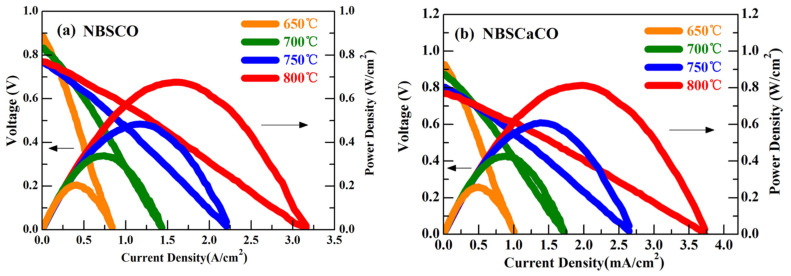
*I*–*V* curves and corresponding output power density of (**a**) NiO–SDC|SDC|NdBa_0.5_Sr_0.5_Co_2_O_5+δ_–SDC and (**b**) NiO–SDC|SDC|NdBa_0.5_Sr_0.25_Ca_0.25_Co_2_O_5+δ_–SDC single cells at various temperatures (adopted with permission from [120]. Copyright 2018, Elsevier).

In [118], a co-doping strategy of both Pr and Ba by Ca in layered PrBaCo_2_O_5+δ_ cobaltite for improving its properties was used. It was shown that this strategy makes it possible to increase electrical conductivity and thermal stability of the samples as well as to reduce ASR values and enlarge MPD (Table 4). The NdBa_0.5_Sr_0.25_Ca_0.25_Co_2_O_5+δ_ cathode demonstrated a very low ASR value of 0.062 Ω cm^2^ at 1073 K and a maximum output power density of 812 mW cm^−2^ at 1073 K (Figure 7). This measurement was much higher than for NdBa_0.5_Sr_0.5_Co_2_O_5+δ_, proving the effectiveness of the co-doping strategy.

Through the electrical conductivity relaxation (ECR) test, the values of chemical bulk diffusion coefficient (*D*_chem_) of oxygen in SmBa_0.6_Sr_0.4_Co_2_O_5+δ_ were measured from 1.63 × 10^−6^ cm^2^ s^−1^ at 773 K to 1.41 × 10^−5^ cm^2^ s^−1^ at 973 K [126]. The temperature dependence of *D*_chem_ in a temperature range of 773–973 K is described by Equation (3):

*D*_chem_ = 1.77 × 10^−5^·exp[−68.039 (kJ mol^−1^)/(*R*·*T*)] (m^2^ s^−1^) (3)

The oxygen transport properties of SmBa_0.5_Sr_0.5_Co_2_O_5+δ_ as a potential cathode material for IT–SOFCs were investigated in [127]. The *D*_chem_ values for SmBa_0.5_Sr_0.5_Co_2_O_5+δ_ were equal to 2.6 × 10^−6^, 9.1 × 10^−6^, and 1.8 × 10^−5^ cm^2^ s^−1^ at 773, 873, and 973 K, respectively. The activation energy of *D*_chem_ within 773–973 K was ~58 kJ mol^−1^. Oxygen permeation flux for the SmBa_0.5_Sr_0.5_Co_2_O_5+δ_ membrane with a thickness of 1.00 mm increased from 0.143 mL min^−1^ cm^−2^ at 773 K to 0.406 mL min^−1^ cm^−2^ at 1073 K under synthetic air at a flow rate of 50 mL min^−1^ and helium at a rate of 25 mL min^−1^. The activation energies of oxygen permeation for a high-temperature region (973–1073 K) and a low-temperature region (773–923 K) were equal to ~24 and 7 kJ mol^−1^, respectively, suggesting that the oxygen diffusion in the high-temperature and low-temperature ranges occurred via surface exchange and bulk diffusion mechanisms, respectively.

### 4.3. B-Site Deficient and B-Site Substituted LnBaCo_2_O_5+δ_ Layered Perovskites

Co-deficient PrBaCo_2−*x*_O_6–δ_ (0 ≤ *x* ≤ 0.1) perovskites were prepared and investigated in [128]. It was found that increasing the concentration of the vacancies in the Co-sublattice ledto an increase in lattice constants and oxygen nonstoichiometry (δ), additionally, the electrical conductivity decreased and electrochemical performance of PrBaCo_2–*x*_O_6–δ_ cathodes improved. The minimum value of ASR and the maximum value of MPD were found for PrBaCo_1.94_O_6–δ_, they were 0.059 Ω cm^2^ at 973 K and 889 mW cm^−2^ at 923 K, respectively. This was 16% lower and higher, respectively, than for the cation-stoichiometric PrBaCo_2_O_6–δ_ phase. The thermal stability of the samples did not change due to the formation of Co-deficiency. Table 5 shows some typical results concerning the electrochemical performance of SOFCs with a B-site substituted REE-barium layered cobaltites as cathodes.

A partial substitution of cobalt in *Ln*BaCo_2_O_5+δ_ perovskites by low-valence Ni- [129,130,131], Zn- [132], or Cu- [86,131,133,134,135] ions led to a slight increase intheir lattice constants [131] and a decrease in oxygen content (δ) [131,132], TEC [129,130,134,135], and electrical conductivity values [86,129,130,131,134]. In some cases, such a substitution improved the electrochemical performance of the derived cathode materials [129,130,132,134]. For Cu-doped SmBa_0.5_Sr_0.5_Co_1.5_Cu_0.5_O_5+δ_ and YBaCoCuO_5+δ_ perovskites, the ASR values were larger and the maximum power density were smaller than for their Cu-free SmBa_0.5_Sr_0.5_Co_2_O_5+δ_ and YBaCo_2_O_5+δ_ counterparts. Moreover, Cu-doped materials showed better long-term stability [86,133,134].

In [136], it was found that a partial substitution of cobalt withhigh-valence tantalum in PrBa_0.94_Co_2_O_5+δ_ stabilizes the A-site-ordered layered perovskite structure, slightly increases TEC and electrical conductivity of the samples, and improves catalytic activity towards ORR. The optimal composition, PrBa_0.94_Co_1.96_Ta_0.04_O_5+δ_, exhibits the lowest polarization resistance (0.020 Ω cm^2^ at 973 K) and the highest power density of 1050 mW cm^−2^ at 973 K and is operated steadily at a loading voltage of 0.6 V over 100 h at 923 K. The PrBa_0.94_Co_1.96_Ta_0.04_O_5+δ_ cathode showed excellent tolerance to CO_2_, as evidenced by its durable polarization resistance of 0.061 Ω cm^2^ at 973 K in air with 10 vol.% of CO_2_. A partial substitution of cobalt by high-valence molybdenum ions in PrBaCo_2−*x*_Mo*_x_*O_5+δ_ almost did not affect the crystal structure of the parent oxide but decreased its TEC and electrical conductivity values [137]. The polarization resistance of the PrBaCo_1.97_Mo_0.03_O_5+δ_ (PBCM–0.03) cathode was 0.067 Ω cm^2^ at 973 K, which was slightly higher than for the pristine PrBaCo_2_O_5+δ_ cathode (PBCO) (0.060 Ω cm^2^ at 973 K). The maximum power density of the single cells containing thePBCM–0.03 and PBCO cathodes at 973 K attained 343 and 339 mW cm^−2^, respectively. It was also found in [137] that trace amounts of high-valence Mo-doping in the PrBaCo_2_O_5+δ_ cathode improved its electrochemical stability. So, generally, the results obtained in [137] showed that PrBaCo_2−*x*_Mn*_x_*O_5+δ_ solid solutions are attractive for applications as SOFCs cathodes.

The isovalent Co-by-Mn substitution in LODPs was studied in [138,139,140,141]. It was found that the TEC and electrical conductivity of SmSrCo_2−*x*_Mn*_x_*O_5+δ_ (0 ≤ *x* ≤ 1) perovskites decreased with increasing *x*. The electrochemical performance of SmSrCo_2_O_5+δ_ slightly decreased after Mn-doping; however, reduced TEC and good chemical compatibility with GDC indicate that these materials may be used as SOFCs cathodes. Similar results were observed in another work [139], where GdBaCo_2–*x*_Mn*_x_*O_5+δ_ (0 ≤ *x* ≤ 2) was formed within the whole studied composition region. Mn-doped oxides showed lower electrical conductivity and increased polarization resistance. Nevertheless, these materials exhibited an increased oxygen content (δ) and reduced TEC values. Moderate TEC values and good catalytic activity in ORR were found for the LnBaCo_2−*x*_Mn*_x_*O_5+δ_ perovskites [140,141].

The effect of the partial substitution of cobalt with iron in layered perovskites of REE and barium and their A-site-deficient and A-site-substituted derivatives was intensively studied in a number of works [86,131,135,142,143,144,145,146,147,148,149,150,151,152,153,154,155,156,157,158]. It was found that Fe-doping results in an increase of lattice constants [131,143,144,145,147,148,151,153,154,157] and oxygen contents [131,146]. Electrical conductivity of such Fe-doped cobaltites decreases with Fe-doping [143,145,146,147,153,154,157]. TECs also decreased [143,147,153], though some works reported an inverse tendency [131,144]. The Fe-doping usually improved the electrochemical performance of the layered cobaltites as SOFCs cathodes, but, in a number of works, increased polarization resistances [144,145] or reduced power densities [142,144] were reported, showing that the final properties of materials depend on both chemical compositions of LODPs and the prehistory of their preparation.

**Figure 8:**
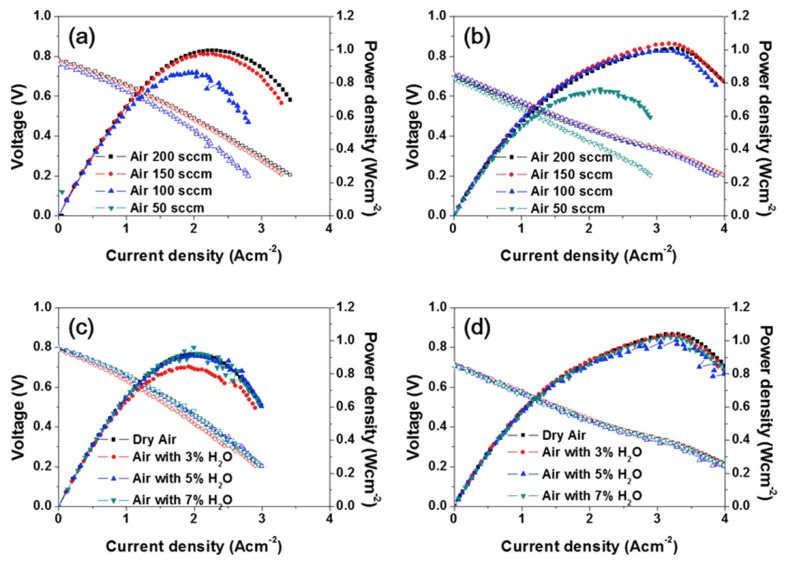
*I–V* curves and corresponding power densities of test cells with different cathodes (**a**,**c**) Ba_0.5_Sr_0.5_Co_0.8_Fe_0.2_O_3–δ_–Nd_0.1_Ce_0.9_O_2–δ_ and (**b**,**d**) NdBa_0.5_Sr_0.5_Co_1.5_Fe_0.5_O_5+δ_–Nd_0.1_Ce_0.9_O_2–δ_ under various cathode air flow rates (**a**,**b**) and humidities (**c**,**d**). The single cells were operated at 923 K with humidified H_2_ (3vol.% H_2_O) as the fuel and ambient air as the oxidant. (**a**,**b**) air flow rate change: 50, 100, 150, and 200 sccm. (**c**,**d**) air humidity change: dry, 3 vol.%, 5vol.%, and 7vol.% H_2_O (reproduced with permission from [151]. Copyright 2016, Elsevier).

Lee et al. [151] compared the electrochemical performance of the Ba_0.5_Sr_0.5_Co_0.8_Fe_0.2_O_3–δ_ single perovskite and the NdBa_0.5_Sr_0.5_Co_1.5_Fe_0.5_O_5+δ_ double perovskite when operated in single cells at different conditions (Figure 8) and demonstrated excellent stability of double perovskite in harsh SOFC environments, including high humidity and low flow rate of air.

In [149,152] it was found that a partial Fe-to-Co substitution in layered cobaltites increases the oxygen-ion diffusion coefficient (Table 6), which should improve the electrochemical activity of these compounds in terms of ORR.

A suspension plasma-sprayed PrBa_0.5_Sr_0.5_Co_1.6_Fe_0.4_O_5+δ_ (PBSCF) cathode operating in a symmetrical cell of PBSCF|ScSZ|PBSCF (ScSZ—scandia stabilized zirconia) and a single cell of Ni–GDC|ScSZ|PBSCF showed good electrochemical performance with as low a ASR value as 0.074 Ω cm^2^ and 0.012 Ω cm^2^at 873 K and 973 K, respectively, peak power densities of 370, 800, and 1350 mW cm^−2^ at 773 K, 873 K, and 973 K, respectively, as well as excellent long-term stability (its polarization resistance remained practically constant during isothermal dwelling at 973 K for 300 h) [154].

In [158], a symmetrical SOFC with LSGM and PrBaCo_0.2_Fe_1.8_O_5+δ_ (PBCF) as a cathode was prepared and tested during its operation with different fuels. According to X-ray diffractometry (XRD) and energy dispersive spectroscopy (EDS) results, the PrBaCo_0.2_Fe_1.8_O_5+δ_ compound had good chemical compatibility with the LSGM electrolyte. At 1073 K, the polarization resistance values of the PBCF symmetrical electrodes were 0.026 and 0.319 Ω cm^2^ in air and H_2_, respectively. The output performances of the electrolyte-supported single cell with the PBFC symmetrical electrodes were 735, 626, and 268 mW cm^−2^ at 1123 K under H_2_, liquefied petroleum gas (LPG), and C_2_H_5_OH fuel, respectively. This cell showed long-term stability at 1023 K for 40 h and 60 h with H_2_ and LPG as the fuel, respectively.

The co-doping strategy was used in [159,160,161] to improve the electrochemical performance of LnBaCo_2_O_5+δ_ cathodes. The XPS results showed that REE and transition metal (TM) ions in PrBaCo_2/3_Fe_2/3_Cu_2/3_O_5+δ_ (PCFC) exist in two different valence states (Pr^3+^/Pr^4+^, Co^3+^/Co^4+^, Fe^3+^/Fe^4+^, and Cu^+^/Cu^2+^). The TEC value of this compound was equal to 16.6 × 10^−6^ K^−1^, which was much smaller than that ofthe unsubstituted layered cobaltite. The polarization resistance values of the PCFC cathode on the SDC and GDC electrolytes were 0.144 and 0.038 Ω cm^2^ at 1073 K, respectively. The maximum power density of a single cell with PCFC on a 300 μm-thick GDC electrolyte reached 659 mW cm^−2^ at 1073 K [159]. Similar results were obtained for the NdBaCo_2/3_Fe_2/3_Cu_2/3_O_5+δ_ (NBCFC) double perovskite, theTEC of which was 15.7 × 10^−6^ K^−1^ within a temperature range of 303–1123 K. The values of the polarization resistance of NBCFC were 0.056 and 0.023 Ω cm^2^ at 1073 K with the Ce_0.9_Gd_0.1_O_1.9_ and La_0.9_Sr_0.1_Ga_0.8_Mg_0.2_O_3–δ_ electrolytes, respectively [160]. Co-substitution with Fe and Mn sharply decreased the TEC from 21.5·10^−6^ K^−1^ for PrBaCo_2_O_5+δ_ to 17.8 × 10^−6^ K^−1^ for PrBaCo_2/3_Fe_2/3_Mn_1/2_O_5+δ_ (PBCFM) at a temperature range of 303–1273 K [161]. When using 300μm-thick Sm_0.2_Ce_0.8_O_1.9_ (SDC) as an electrolyte, the ASR and maximum power density values were equal to 0.028 Ω cm^2^ and 588 mW cm^−2^ at 1073 K, respectively. The SDC-impregnated PBCFM composite cathode showed improved electrochemical characteristics; its ASR and peak power density were 0.23 Ω cm^2^ and 621 mW cm^−2^ at 1073 K, respectively.

### 4.4. Composites Based on LnBaCo_2_O_5+δ_ Layered Perovskites

Composites of layered cobaltites with different solid electrolytes were extensively studied as possible cathode materials for SOFCs based on both oxygen-ion [87,162,163,164,165,166,167,168,169] and proton-conducting solid electrolytes (SE) [170,171], as well as oxygen separation membranes [172,173]. The addition of SE to the layered cobaltites lowers their TEC values [87,162,164,165,166], making them more chemically and thermomechanically compatible with electrolytes; it also considerably improves the electrochemical performance of composite cathodes in comparison with the single-phase ones due to the enlarging active zones at which ORR can occur [162,163,165,166,168,169,170,171].

The addition of 20 wt.% of Bi_2_O_3_ to LaBaCo_2_O_5+δ_ resulted in the lowest ASR value (0.020 Ω cm^2^ at 1073 K in air), which was about a seventh of that of the LaBaCo_2_O_5+δ_ cathode in the same conditions [163]. At a current density of 0.2 A cm^−2^, the cathodic overpotential of LaBaCo_2_O_5+δ_+20 wt.%Bi_2_O_3_ was approximately 12.6 mV at 973 K, while the corresponding value for LaBaCo_2_O_5+δ_ was 51.0 mV.

**Figure 9:**
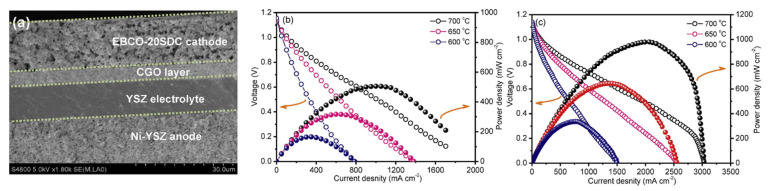
(**a**) Cross-section view of SEM micrograph of the Ni–YSZ|YSZ|CGO|EBCO–20SDC cell. The *V*–*I* and *P*–*I* curves of the Ni–YSZ|YSZ|CGO|EBCO–*x*SDC cells from 873 K to 973 K at (**b**) *x* = 0 and (**c**) *x* = 20 (adopted with permission from [165]. Copyright 2019, Elsevier).

In [165], the EuBa_0.98_Co_2_O_5+δ_+*x* wt.%Ce_0.9_Sm_0.1_O_1.9_ (EBCO–*x*SDC) (0 ≤ *x* ≤ 40) composites were systematically studied as cathode materials for SOFCs. It was found that EBCO–*x*SDC materials had excellent electrocatalytic ORR activity due to the synergistic effects of the high electronic-conducting EBCO phase and ionic-conducting SDC electrolyte. The best cathode performance among the studied composites was exhibited by the EBCO–20SDC material (0.028 Ω cm^2^ at 973 in air), when tested in a single-cell Ni–YSZ|YSZ|CGO|EBCO–20SDC (Figure 9). A high power density of 980 mW cm^−2^ at 973 K was achieved, which was approximately two times higher than that for the EBCO cathode-based fuel cell. The results of electrochemical impedance spectroscopy (EIS) showed that the charge transfer was the rate-limiting step at the cathode interface. The addition of SDC to the EBCO improved both the charge transfer reaction and the gas diffusion process due to the high oxygen-ion conductivity and large surface of the SDC electrolyte.

Idrees et al. [168], using a facile and effective one-pot sol-gel method, prepared a PrBa_0.92_Co_2_O_6–δ_–40 wt.%Ce_0.8_Sm_0.2_O_1.9_ (OPCC) composite cathode material and comparatively studied its electrochemical performance in tandem with a composite cathode with the same composition which was synthesized by means of a traditional ball-milling method (BMCC), as well as with the single-phase PrBa_0.92_Co_2_O_6–δ_ cathode. Among the three studied cathodes, OPCC showed the lowest ASR (0.015 Ω cm^2^ at 1023 K), indicating the highest ORR catalytic activity. The OPCC-based anode-supported single cell demonstrated the highest peak power densities, with a typical value of 1011 mW cm^−2^ at 1023 K in contrast to 783 mW cm^−2^ for the BMCC-based cell and 574 mW cm^–2^ for the PrBa_0.92_Co_2_O_6–δ_–based cell. The OPCC-based cell also showed stable performance without obvious degradation over 100 h at 973 K.

In [169], the Pr_0.95_BaCo_2_O_6–δ_–*x*Ce_0.8_Sm_0.2_O_1.9_ (*x* = 0, 30, 40, 50) composites (PBCO–*x*SDC) were successfully prepared and investigated. It was found that the addition of SDC to the Pr^3+^-deficient perovskite decreased its TEC and electrical conductivity values but enhanced its catalytic activity over ORR. The best electrochemical performance was shown by the PBCO–40SDC cathode, for which ASR was 0.005 Ω cm^2^ at 1023 K. An anode-supported single cell with this cathode demonstrated high peak power densities, such as 1171 mW cm^−2^ at 1023 K and 917 mW cm^−2^ at 973 K.

Electrical conductivity and oxygen permeability of the Ce_0.8_Gd_0.2_O_2–δ_–GdBaCo_2_O_5+δ_ (CGO–GBCO) and Ce_0.8_Gd_0.2_O_2–δ_–PrBaCo_2_O_5+δ_ (CGO–PBCO) dual-phase composites were studied in [172,173]. The thermally activated oxygen permeation flux reached 0.28 mL min^−1^ cm^−2^ at air/He condition for the 0.62 mm-thick CGO–GBCO specimen at 1223 K, which was an order of magnitude larger than that of the GBCO specimen at the same conditions [172]. For the CGO–PBCO (6/4) membrane with 0.6 mm in thickness, oxygen flux was as large as 2.38·10^−7^ mol cm^−2^ s^−1^ at 1198 K [173]. It was found that the CGO and PBCO phases exhibited good chemical compatibility and structural stability.

In [174], the Nd_1–*x*_BaCo_2_O_5+δ_+*x*/2Bi_2_O_3_ (*x* = 0.05, 0.1) composites were synthesized via a glycine–nitrate process. The addition of bismuth oxide to the Nd^3+^-deficienced ceramics effectively increased their electrical conductivity and reduced TEC. Polarization resistance and the maximum power density of the Nd_0.95_BaCo_2_O_5+δ_+0.125Bi_2_O_3_ composite cathode at 1073 K were 0.026 Ω cm^2^ and 720 mW cm^−2^, respectively.

NdSrCo_2_O_5+δ_ (NSCO) perovskite was used as a cathode material for the Ce_0.8_Gd_0.2_O_2–δ_ (GDC)-supported microtubular solid oxide fuel cells (MT–SOFCs) [175]. The MT–SOFC with an outer tube diameter of 1.86 mm, an electrolyte thickness of 180 μm, and an NSCO–GDC (1:1) composite cathode presented the best electrochemical performance. The flexural strength of this cell was 177 MPa, ohmic and polarization resistance values of the cell were 0.15 and 0.03 Ω cm^2^ at 1073 K, and its maximum power density reached 0.67 W cm^−2^ at 1073 K.

### 4.5. LnBaMe’Me”O_5+δ_ Layered Perovskites and Their Solid Solutions

*Ln*Ba*Me*’*Me*”O_5+δ_ are LODPs, the B-sites of which are occupied by two transition metals (usually 3*d*-metals) taken in the same quantities. These phases, similar to layered cobaltites of REE and barium, were intensively studied as possible cathode materials for SOFCs [176,177,178,179,180,181,182,183,184,185,186,187,188,189,190]. Some typical results demonstrating their electrochemical performance are collected in Table 7. As can be seen from these data, *Ln*Ba*Me*’*Me*”O_5+δ_ phases may be used as SOFCs cathodes with both oxygen-ion conducting (Ce_0.8_Sm_0.2_O_1.9_, La_0.9_Sr_0.1_Ga_0.8_Mg_0.2_O_3–δ_, and Gd_0.1_Ce_0.9_O_1.95_) and proton-conducting (BaZr_0.1_Ce_0.7_Y_0.2_O_3–δ_) solid electrolytes; at the same time, they demonstrate high electrochemical activity in ORR, which is close to the activity of layered REE–barium cobaltites and their derivatives. The electrochemical performance of *Ln*Ba*Me*’*Me*”O_5+δ_ compounds may be improved by the addition of solid electrolytes with the formation of composites [182,185,186], partial cation substitution [187,190], or creation of A-site deficiency [188].

The GdBaFeNiO_5+δ_ material exhibits good chemical compatibility with the Sm_0.2_Ce_0.8_O_1.9_ electrolyte at temperatures below 1273 K; its ASR value is 0.219 Ω cm^2^ at 1073 K, and the maximum power density of the Ni–Sm_0.2_Ce_0.8_O_1.9_|Sm_0.2_Ce_0.8_O_1.9_|GdBaFeNiO_5+δ_ single cell reaches 287 mW cm^−2^ at 1073 K [185]. The activity and performance of the GdBaFeNiO_5+δ_ cathode can be improved by the impregnation of nano-sized Sm_0.2_Ce_0.8_O_1.9_ particles: the polarization resistance is decreased by more than 14 times (down to 0.065 Ω cm^2^ at 1073 K), and the maximum power density of the single cell increased by 1.9 times (up to 515 mW cm^−2^ at 1073 K).

The La_1.4_Ca_0.6_CoMnO_5+δ_ (LCCM) material has a monoclinic structure, high structural stability up to 1173 K, and exhibits good chemical compatibility with the La_0.9_Sr_0.1_Ga_0.8_Mg_0.2_O_3–δ_ (LSGM) and Sm_0.2_Ce_0.8_O_1.9_ (SDC) electrolytes at temperatures of up to 1273 K. The maximum power density of a NiO–SDC|SDC|LSGM|LCCM single cell reaches 445 mW cm^−2^ at 1073 K [186]. The electrochemical performance, thermal expansion behavior, and stability of LCCM improve by adding appropriate amounts of SDC. The LCCM–30 wt.%SDC composite cathode shows the increased electrochemical performance: the ASR is decreased by 68% and the maximum power density is increased by 22%.

**Figure 10:**
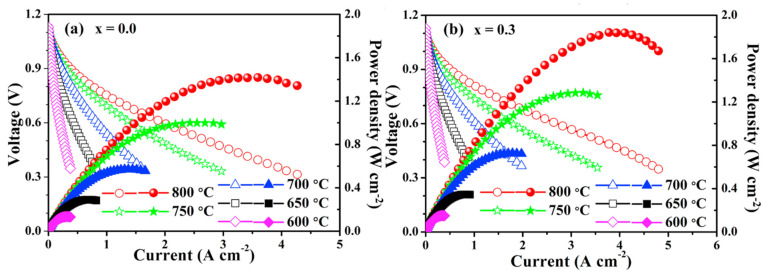
*I*–*V* and *I*–*P* curves of NdBa_1–*x*_Ca*_x_*CoCuO_5+δ_ based single cells at (**a**) *x* = 0 and (**b**) *x* = 0.3 (adopted with permission from [187]. Copyright 2018, Elsevier).

A partial substitution of Ba with Ca in NdBaCoCuO_5+δ_ resulted in decreased TEC and oxygen content and increased the electrical conductivity in air [187]. Compared to the parent oxide, the modified sample has a greatly enhanced electrochemical performance. The ASR of NdBa_1−*x*_Ca*_x_*CoCuO_5+δ_-based symmetrical cells with a Gd_0.1_Ce_0.9_O_1.95_ electrolyte at 973 K dropped from 0.062 Ω cm^2^ for *x* = 0 to 0.038 Ω cm^2^ for *x* = 0.3. The maximum power density for NdBa_1−*x*_Ca*_x_*CoCuO_5+δ_-based single cells at 1073 K increased from 1420 mW cm^−2^ for *x* = 0 to 1840 mW cm^−2^ for *x* = 0.3 (Figure 10).

The Y_1–*x*_BaCoCuO_5+δ_ (*x* = 0.00, 0.03, 0.05, 0.07, 0.10) layered perovskites with a Y^3+^-deficiency were studied as cathodes for SOFCs in work [188]. These compounds crystallize in orthorhombic syngony, and their lattice constants increase with an increasing Y^3+^ deficiency. The oxygen content and electrical conductivity values of Y_1−*x*_BaCoCuO_5+δ_ decrease as *x* increases. The results of EIS studies indicate that the creation of a Y^3+^deficiency reduces the polarization resistance, the lowest value of which was 0.029 Ω cm^2^ at 1073 K and was observed for the Y_0.93_BaCoCuO_5+δ_ sample. The LSGM electrolyte-supported single cell with the Y_0.93_BaCoCuO_5+δ_ cathode demonstrated the peak power density values of 862, 643, 426, 274, and 152 mW cm^−2^ at 1123, 1073, 1023, 973, and 923 K, respectively.

### 4.6. The Other Layered Oxygen-Deficient Double Perovskites

Layered double cobaltites usually display high electronic and ionic conductivity, as well as high electrocatalytic activity in the ORR, but they have some drawbacks, such as high TEC values (providing relatively low thermomechanical compatibility of layered cobaltites and typical solid electrolytes in SOFCs), high cost, etc. [191]. These disadvantages are overcome in Co-free perovskite cathodes, including double perovskites, such as GdBaFeNiO_5+δ_ [183,185] and layered ferrites of REEs and barium [89,158,192,193,194,195,196,197]. The main advantage of layered ferrites is their high stability levels in both oxidizing and reducing atmospheres [4,191]; this makes them promising electrode materials for A–SOFCs [89,192,193,194,196], and S–SOFCs [158,195,197] based on oxygen-ion-conducting [158,194,195,196] and proton-conducting [89,193,197] electrolytes.

The electrical conductivity and thermal expansion of *Ln*BaFe_2_O_5+δ_ (*Ln* = La, Pr, Nd, Sm, Gd, and Y)-layered ferrites decreased at smaller *Ln*^3+^ ionic radii; for example,TEC values within 293–1173 K temperature intervals decreased from 19.4 × 10^−6^ K^−1^ for *Ln* = La to 14.6 × 10^−6^ K^−1^ for *Ln* = Y [192]. The lowest polarization resistance in air under an open circuit voltage was found for the SmBaFe_2_O_5+δ_ electrode (YSZ as the electrolyte): 0.043 Ω cm^2^ at 1073 K. The single cell with this material serving as its cathode delivered the highest peak power density of 1026 mW cm^−2^ at 1073 K [192]. The LaBa_0.5_Sr_0.5_Fe_2_O_6–δ_ cathode showed a low polarization resistance of 0.152 Ω cm^2^ at 1023 K and a maximum power density of 370 mW cm^−2^ in a LaBa_0.5_Sr_0.5_Fe_2_O_6–δ_|SDC|LaBa_0.5_Sr_0.5_Fe_2_O_6–δ_ S–SOFC [194].

**Figure 11:**
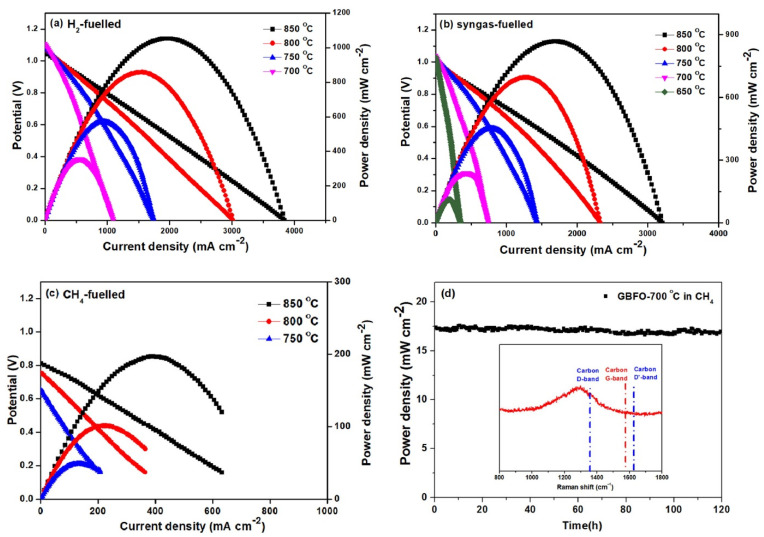
Performance of the single cell with GdBaFe_2_O_5+δ_ as its symmetrical electrodes for different fuels: (**a**) H_2_, (**b**) humidified syngas, and (**c**) humidified CH_4_. (**d**) Electrochemical stability test of the cell with humidified CH_4_ as a fuel during a 120 h operation. Inset: Raman spectrum of the anode surface area after stability test (reproduced with permission from [195]. Copyright 2021, Elsevier).

Figure 11 shows the electrochemical performance of the S–SOFC with a GdBaFe_2_O_5+δ_|La_0.9_Sr_0.1_Ga_0.8_Mg_0.2_O_3–δ_|GdBaFe_2_O_5+δ_ configuration when using dry H_2_, humidified syngas (61% H_2_, 24% CH_4_, 9.3% CO, 3.4% N_2_, 2.3% CO_2_, and 5 ppm H_2_S), and humidified CH_4_ (3% H_2_O) as the fuels and the ambient air as the oxidant. The maximum peak power density of this cell at 1125 K was 1053, 868, and 197 mW cm^−2^ for the different fuels, respectively. The observed results indicate that this cell can efficiently operate with complex hydrocarbons fuels. This cell was also tested under a constant potential of 0.35 V with CH_4_ as fuel at 973 K for 120 h to assess the carbon tolerance of the GdBaFe_2_O_5+δ_ anode and the stability of the cell (Figure 11d). No degradation of the cell performance was observed. The Raman spectrum (inset in Figure 11d) shows no peaks corresponding to the carbon deposited on the GdBaFe_2_O_5+δ_ anode surface, indicating that this material has a high coking tolerance. The peak at ca. 1300 cm^−1^ was assigned to FeOOH and was formed because a small part of exsolved iron nanoparticles reacted with water in humidified CH_4_.

The oxygen transport properties and the chemical stability of PrBaFe_2_O_5+δ_ (PBF)-layered double perovskite were systematically studied in [198]. The oxygen permeation flux of 0.7 mm-thick samples and the oxygen-ion conductivity were 4.7 × 10^–1^ mL min^−1^ cm^−2^ and 0.12 Scm^−1^ at 1173 K, respectively. The characteristic thickness estimated from the membrane and conductivity relaxation tests was ~0.6 mm at 1173 K. The PBF oxide exhibited superior chemical stability in CO_2_-containing atmosphere.

Layered manganites of barium and REEs (for example, PrBaMn_2_O_5+δ_) have been considered as possible electrode materials both for A–SOFCs and S–SOFCs, as well as for SOECs cathodes [199,200,201]. The high thermal stability of Fe-doped PrBaMn_2–*x*_Fe*_x_*O_6–δ_ perovskites both in oxidizing and reducing atmospheres and the moderate TEC values make these phases good candidates for electrochemical applications [200]. These phases may exchange relatively large amounts of oxygen with the atmosphere, which makes them promising oxygen storage materials as well.

The Sr-deficient Sr_1.9_FeNb_0.9_Mo_0.1_O_6–δ_ double perovskite was prepared via a solid state reaction method and tested as the electrode in S–SOFCs [202]. The electrocatalytic activity of Sr_1.9_FeNb_0.9_Mo_0.1_O_6–δ_ was greatly improved by the impregnation of the solution of Pd(NO_3_)_2_ to form Pd–Sr_1.9_FeNb_0.9_Mo_0.1_O_6–δ_ composite electrodes. The single cell with such symmetrical electrodes after two-time impregnation achieved the peak power densities of 935.4, 196.5, and 11.2 mW cm^−2^ at 1123 K in dry H_2_, humidified CH_4_, and 17CH_4_–83CO_2_ mixed gas, respectively, revealing superior performance in different fuels.

In [90], it was shown that Sr_2_Co_1−*x*_Nb*_x_*FeO_5+δ_ (*x* = 0.0–0.2) double perovskites have good structural stability and chemical compatibility with La_0.9_Sr_0.1_Ga_0.8_Mg_0.2_O_3–δ_ and Ce_0.8_Sm_0.2_O_1.9_ electrolytes. The ASR of the Sr_2_Co_1.9_Nb_0.1_FeO_5+δ_ cathode was 0.081 Ω cm^2^ at 973 K on the LSGM electrolyte. This cathode showed good electrocatalytic activity for ORR, functional stability, and high electrical conductivity.

The possibility of using Sr_2_Sc_0.1_Nb_0.1_Co_1.5_Fe_0.3_O_6–2δ_ thin films as cathodes for IT–SOFCs was tested in [203]. It was found that the film grown along the [110] direction on the YSZ substrate demonstrated lower polarization resistances and smaller activation energy than the film grown along the [100] direction on the SDC/YSZ substrate, indicating better ORR activity. It was also found that the lower Sr-enrichment and higher cobalt-ion oxidation states were beneficial for the ORR.

The La-deficient La_2−*x*_CoTiO_6–δ_ (0 ≤ *x* ≤ 0.20) double perovskites were chemically stable under oxidizing conditions towards CGO, whereas they reacted with YSZ [204]. The La_2−*x*_CoTiO_6–δ_/Ce_0.8_Gd_0.2_O_1.9_ composites were studied as electrodes of symmetrical cells. The lowest polarization resistance of 0.39 Ω cm^2^ at 1073 K was found for materials with *x* = 0.05.

### 4.7. Short Resume

Layered oxygen-deficient double perovskites (primarily cobaltites and their derivatives) are promising cathode materials for IT–SOFCs due to their high electrocatalytic activity in ORR, stability at elevated temperatures and chemical and thermomechanical compatibility with solid electrolytes.

Despite numerous advantages, LODPs have a number of drawbacks in terms of their applications in SOFCs. The literature analyses performed above shows that the main problems are their degradation in CO_2_-containing atmospheres and relatively large TEC values, as well as chemical expansion at high temperatures due to the loss of weakly-bonded oxygen.

**Figure 12:**
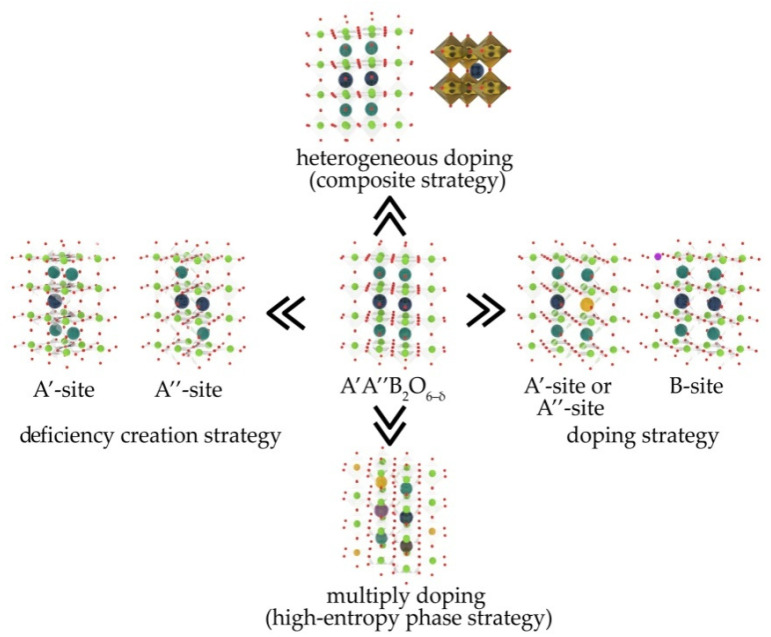
Different strategies of improving the performance of A’A”B_2_O_6–δ_ layered oxygen deficient double perovskites.

These disadvantages can be eliminated or reduced by tuning the chemical composition of the LODPs or by producing their composites containing different solid electrolytes as a second phase. The electrochemical performance of LODPs can be essentially improved both by the substitution of cations in their A- or/and B-sublattices or by the creation of small cation deficiencies in the A-sublattice (Figure 12). The TEC values and chemical expansion of the LODPs may be reduced by the formation of high-entropy oxides (HEOs) on their base, in which A- or/and B-positions are shared by at least five REEs (as well as alkaline–earth elements) and transition metals, respectively, in equal or near-equal atomic ratios. Such HEOs should be more stable and possess lower polarization resistances compared when with other cathodes based on LODPs. The addition of solid electrolytes to LODPs lowers their TECs and makes them more chemically and thermomechanically compatible with electrolytes and can also improve the electrochemical performanceof composite cathodes due to enlarging the zones of the ORRs. The creation of a hierarchical porous microstructure of cathodes also enlarges the areas of the ORRs and improves their electrochemical and electrocatalytical performance.

LODPs based on light REEs have small oxygen deficiencies (large amounts of weakly-bonded oxygen), high values of the Seebeck coefficient, and electrical conductivity, and they also contain transition metal ions in different oxidation states. For these reasons they may be used as high-temperature thermoelectrics, oxygen storage materials, or photocatalysts for the oxidation of organic substances.

## 5. Conclusions

Layered oxygen-deficient perovskites (LODPs) are considered promising candidates for use as cathodes in IT–SOFC applications. In this review, we shortly summarized the available literature data concerning their crystal structure, thermal, and electrotransport and functional, especially electrochemical, properties. The phase transitions of different natures (structural, electric, and magnetic), which take place in LODPs due to the variations of temperature and their chemical composition, were also discussed. The electrochemical performance of materials belonging to different groups of LODPs (cobaltites, ferrites, etc.) in single cells based on both oxygen-ion- and proton-conducting solid electrolytes was found to be quite high for applied purposes. The main focus was on various chemical engineering approaches to improve the electrochemical activity of these materials (creation of cationic deficiencies, doping on different sites, modification by noble metals and solid electrolytes nanoparticles, etc.). It was demonstrated that some LODPs can effectively operate as electrodes of symmetrical SOFCs fueled with hydrogen, methane, or complex hydrocarbons. The other possible areas of usage of these complex oxides (high-temperature thermoelectrics, oxygen storage materials, photocatalysts, etc.) were alsofinally depicted.

Although this work overviews the last trends in LODPs, a number of recent publications fell within the reviewing procedure. In our opinion, these works [205,206,207,208,209,210,211] should be mentioned with no detailed analysis.

## Figures and Tables

**Table 1:** TEC values of some layered oxygen-deficient perovskites.

Compound (Space Group)	TEC 10^6^, K^−1^	Temperature Interval, K	Refs.
NdBaCoFeO_5+δ_ (*P*4/mmm)	16.6	300–653	[62]
	26.5	653–1100	
SmBaCoFeO_5+δ_ (*P*4/*mmm*)	13.6	300–518	[62]
	19.3	518–1100	
GdBaCoFeO_5+δ_ (*P*4/*mmm*)	12.9	300–553	[62]
	19.9	553–1100	
SmBaCo_2_O_5+δ_ (*Pmmm*)	21.2	293–1173	[63]
SmBaCoCuO_5+δ_ (*Pmmm*)	15.0	293–1173	[63]
NdBaCo_2_O_5+δ_ (*P*4/*mmm*)	19.7	293–1173	[63]
NdBaCoCuO_5+δ_ (*P*4/*mmm*)	16.5	293–1173	[63]
NdBaCo_2_O_5+δ_ (*P*4/*mmm*)	18.3	300–530	[64]
	23.8	530–1300	
NdBaCo_1.8_Fe_0.2_O_5+δ_ (*P*4/*mmm*)	18.8	300–530	[64]
	21.9	530–1300	
NdBaCo_1.6_Fe_0.4_O_5+δ_ (*P*4/*mmm*)	18.9	300–530	[64]
	21.9	530–1300	
NdBaCo_1.4_Fe_0.6_O_5+δ_ (*P*4/*mmm*)	18.3	300–530	[64]
	22.1	530–1300	
NdBaCo_1.2_Fe_0.8_O_5+δ_ (*P*4/*mmm*)	18.4	300–530	[64]
	21.9	530–1300	
PrBaCo_2_O_6–δ_ (*Pmmm*)	16.8	298–473	[66]
	21.6	473–1273	
NdBaCo_2_O_6–δ_ (*Pmmm*)	16.3	298–473	[66]
	21.6	473–1273	
NdBaCo_2_O_6–δ_ (*Pm*3*m*)	11.9	298–473	[66]
	22.6	473–1273	

**Table 2:** CEC values of some layered oxygen-deficient perovskites.

Compound (Space Group)	CEC 10^3^	Direction	Refs.
NdBaCoFeO_5+δ_ (*P*4/*mmm*)	11.04	in-plane	[62]
	−17.27	out-of-plane	
	4.59	volume	
SmBaCoFeO_5+δ_ (*P*4/*mmm*)	20.72	in-plane	[62]
	−18.00	out-of-plane	
	23.94	volume	
GdBaCoFeO_5+δ_ (*P*4/*mmm*)	17.78	in-plane	[62]
	−12.36	out-of-plane	
	25.97	volume	
LaBaCuFeO_5+δ_ (*Pm*3*m*)	9.20	volume	[78]
LaBa_0.75_Sr_0.25_CuFeO_5+δ_ (*Pm*3*m*)	7.55	volume	[78]
PrBaCuFeO_5+δ_ (*P*4/*mmm*)	8.72	volume	[78]
LaBaCoFeO_5+δ_ (*Pm*3*m*)	17.3	volume	[79]
LaBaCoCuO_5+δ_ (*Pm*3*m*)	15.7	volume	[79]
PrBaCuFeO_5+δ_ (*P*4/*mmm*)	12.0	volume	[80]

**Table 3:** Performance (ASR: area specific resistance, MPD: maximal power density) of SOFCs based on REE–barium layered double cobaltites.

Cathode	Electrolyte	ASR, Ω cm^2^ (*T*, K)	MPD, mW cm^−2^ (*T*, K)	Refs.
GdBaCo_2_O_5+δ_	Ce_0.2_Gd_0.2_O_2–δ_	0.534 (918)	–	[91]
GdBaCo_2_O_5+δ_	YSZ	0.25 (998)	250 (1073) 500 (1073) *	[92]
PrBaCo_2_O_5+δ_	GDC	0.23 (873)	–	[93]
LaBaCo_2_O_5+δ_	LSCM	–	516 (1073)	[94]
GdBaCo_2_O_5+δ_	LSCM	–	443 (1073)	[94]
PrBaCo_2_O_5+δ_	SDC	–	866 (923)	[95]
NdBaCo_2_O_5+δ_	SDC	0.08 (973)	–	[96]
YBaCo_2_O_5+δ_	YSZ	2.03 (1053)	–	[97]
PrBaCo_2_O_5+δ_	BaCe_0.5_Zr_0.3_Y_0.16_Zn_0.04_O_3–δ_	0.12 (973)	361 (973)	[98]
LaBaCo_2_O_5+δ_	Gd_0.1_Ce_0.9_O_1.95_	0.0086 (1073)	–	[99]

* With an intermediate porous YSZ layer introduced between the solid electrolyte and the cathode.

**Table 4:** Performance (ASR: area specific resistance, MPD: maximal power density) of SOFCs based on A-site deficient and A-site substituted REE-barium layered double cobaltites.

Cathode	Electrolyte	ASR, Ω cm^2^ (*T*, K)	MPD, mW cm^−2^ (*T*, K)	Refs.
Pr_0.95_BaCo_2_O_5+δ_	Ce_0.9_Gd_0.1_O_1.95_	0.054 (923)	–	[104]
Nd_0.96_BaCo_2_O_5+δ_	Ce_0.9_Gd_0.1_O_1.95_	0.043 (973)	600 (973)	[105]
Sm_0.95_BaCo_2_O_5+δ_	GDC	0.038 (1023)	–	[106]
Sm_0.90_BaCo_2_O_5+δ_	Ce_0.9_Gd_0.1_O_2–δ_	0.035 (973)	–	[107]
LaBa_0.90_Co_2_O_5+δ_	Ce_0.9_Gd_0.1_O_1.95_	0.023 (973)	–	[108]
PrBa_0.92_Co_2_O_5+δ_	Ce_0.9_Gd_0.1_O_1.95_	0.093 (873)	–	[109]
PrBa_0.94_Co_2_O_5+δ_	Ce_0.9_Gd_0.1_O_1.95_	0.042 (873)	1030 (973)	[110]
NdBa_0.90_Co_2_O_5+δ_	Ce_0.9_Gd_0.1_O_2_	0.1 (973)	–	[111]
Pr_0.5_Ba_0.245_Ca_0.25_CoO_3–δ_	Ce_0.9_Gd_0.1_O_1.95_	–	2080 (1073)	[112]
PrBa_0.42_Sr_0.5_Co_2_O_5+δ_	La_0.8_Sr_0.2_Ga_0.8_Mg_0.2_O_3–δ_	0.082 (1023)	–	[113]
PrBa_0.5_Sr_0.5_Co_2_O_5+δ_	Ce_0.9_Gd_0.1_O_2–δ_	0.286 (873)	–	[117]
Pr_0.9_Ca_0.1_Ba_0.8_Ca_0.2_Co_2_O_5+δ_	SDC	0.069 (973)	712 (1073)	[118]
NdBa_0.5_Sr_0.5_Co_2_O_5+δ_	SDC	0.09 (1073)	341 (1073)	[119]
NdBa_0.5_Sr_0.25_Ca_0.25_Co_2_O_5+δ_	Sm_0.2_Ce_0.8_O_1.9_	0.062 (1073)	812 (1073) *	[120]
NdBa_0.75_Ca_0.25_Co_2_O_5+δ_	GDC	0.066 (873)	2114 (873)	[121]
GdBa_0.5_Sr_0.5_Co_2_O_5+δ_	BaCe_0.5_Zr_0.3_Y_0.16_Zn_0.04_O_3–δ_	0.15 (973)	350 (973)	[122]
YBa_0.5_Sr_0.5_Co_2_O_5+δ_	La_0.9_Sr_0.1_Ga_0.8_Mg_0.115_Co_0.085_O_2.85_	–	650 (1073)	[123]
YBa_0.8_Sr_0.2_Co_2_O_5+δ_	Ce_0.9_Gd_0.1_O_2–δ_	0.21 (973)	–	[124]
Pr_0.5_Sm_0.5_Ba_0.5_Sr_0.5_Co_2_O_5+δ_	Ce_0.9_Gd_0.1_O_2_	0.10 (973)		[125]

* For the single cell containing the composite NdBa_0.5_Sr_0.25_Ca_0.25_Co_2_O_5+δ_–Sm_0.2_Ce_0.8_O_1.9_ cathode.

**Table 5:** Performance (ASR: area specific resistance, MPD: maximal power density) of SOFCs based on B-site substituted REE–barium layered double cobaltites.

Cathode	Electrolyte	ASR, Ω cm^2^ (*T*, K)	MPD, m Wcm^−2^ (*T*, K)	Refs.
PrBaCo_1.6_Ni_0.4_O_5+δ_	Ce_0.8_Sm_0.2_O_1.9_	0.018 (1073)	732 (1073)	[129]
PrBa_0.5_Sr_0.5_Co_1.9_Ni_0.1_O_5+δ_	YSZ	0.297 (1073)	120 (1073)	[130]
PrBa_0.9_Ca_0.1_Co_1.85_Zn_0.15_O_5+δ_	BZCYYb	0.04 (1023) *	876 (1023) *	[132]
SmBa_0.5_Sr_0.5_Co_1.5_Cu_0.5_O_5+δ_	GDC	0.201 (873)	1760 (923)	[133]
YBaCo_1.4_Cu_0.6_O_5+δ_	La_0.9_Sr_0.1_Ga_0.8_Mg_0.2_O_3–δ_	0.076 (1023)	815 (1123)	[134]
YBaCoCuO_5+δ_	La_0.9_Sr_0.1_Ga_0.8_Mg_0.2_O_3–δ_	0.138 (973)	543 (1073)	[86]
PrBa_0.94_Co_1.96_Ta_0.4_O_5+δ_	GCO	0.020 (973)	1050 (973)	[136]
PrBaCo_1.97_Mo_0.03_O_5+δ_	Sm_0.2_Ce_0.8_O_1.9_	0.067 (973)	339 (973)	[137]
SmSrCo_0.8_Mn_0.2_O_5+δ_	Ce_0.9_Gd_0.1_O_1.95_	0.078 (973)	–	[138]
GdBaCo_1.8_Mn_0.2_O_5+δ_	Ce_0.9_Gd_0.1_O_2–δ_	0.078 (92)	–	[139]
GdBaCo_1.5_Mn_0.5_O_5+δ_	LSGM	0.040 (1123)	–	[140]
PrBaFe_2_O_5+δ_	Sm_0.2_Ce_0.8_O_1.9_	0.18 (973)	–	[143]
YBaCo_1.8_Fe_0.2_O_5+δ_	La_0.8_Sr_0.2_Ga_0.8_Mg_0.115_Co_0.085_O_2.85_	0.13 (973)	768 (1073)	[145]
PrBaCo_1.6_Fe_0.4_O_5+δ_	Ce_0.8_Sm_0.2_O_2–γ_	0.13 (973)	446.4 (973)	[145]
PrBaCoFeO_5+δ_	YSZ	–	720 (973) **	[146]
PrBaCo_1.5_Fe_0.5_O_5+δ_	La_0.8_Sr_0.2_Ga_0.83_Mg_0.17_O_2.815_	0.07 (1123)	697 (1123)	[147]
NdBa_0.5_Sr_0.5_Co_1.5_Fe_0.5_O_5+δ_	Nd_0.1_Ce_0.9_O_2–δ_	–	1010 (923) ^#^	[151]
GdBaCo_1.4_Fe_0.6_O_5+δ_	Ce_0.9_Gd_0.1_O_2–δ_	0.096 (923)	–	[153]
PrBa_0.5_Sr_0.5_Co_1.8_Fe_0.2_O_5+δ_	ScSZ	0.012 (973)	1350 (973)	[154]
NdBaCo_1.6_Fe_0.4_O_5+δ_	Gd_0.1_Ce_0.9_O_2_	0.17 (973)	–	[155]
NdBa_0.9_Co_1.9_Fe_0.1_O_5+δ_	Ce_0.9_Gd_0.1_O_2–δ_	0.14 (973)	–	[157]
PrBaCo_0.2_Fe_1.8_O_5+δ_	La_0.9_Sr_0.1_Ga_0.8_Mg_0.2_O_3_	–	735 (1123)	[158]
PrBaCo_2/3_Fe_2/3_Cu_2/3_O_5+δ_	Ce_0.9_Gd_0.1_O_1.95_	0.038 (1073)	659 (1073)	[159]
NdBaCo_2/3_Fe_2/3_Cu_2/3_O_5+δ_	La_0.9_Sr_0.1_Ga_0.8_Mg_0.2_O_3–δ_	0.023 (1073)	719 (1073)	[160]
PrBaCo_2/3_Fe_2/3_Mn_1/2_O_5+δ_	Sm_0.2_Ce_0.8_O_1.9_	0.028 (1073)	588 (1073)	[161]

* For the composite PrBa_0.9_Ca_0.1_Co_1.85_Zn_0.15_O_5+δ_+BZCYYb (6:4 weight ratio) cathode. ** For the composite PrBaCoFeO_5+δ_+YSZ. ^#^ For the composite NdBa_0.5_Sr_0.5_Co_1.5_Fe_0.5_O_5+δ_+Nd_0.1_Ce_0.9_O_2–δ_.

**Table 6:** Oxygen anion diffusion coefficients and activation energies calculated using molecular dynamics for REE–barium layered double cobaltites.

Material Composition	Diffusion Coefficient (cm^2^ s^−1^)	*T*, K	Activation Energy (kJ mol^−1^)	Refs.
PrBaCo_2_O_5+δ_	3.0·10^−8^	873	40.5	[149]
PrBa_0.5_Sr_0.5_Co_2_O_5+δ_	8.33·10^−8^	873	28.9	[149]
PrBaCo_1.5_Fe_0.5_O_5+δ_	8.0·10^−8^	873	24.12	[149]
PrBa_0.5_Sr_0.5_CoFeO_5+δ_	5.50·10^−8^	873	41.6	[152]
PrBa_0.5_Sr_0.5_Co_1.5_Fe_0.5_O_5+δ_	1.18·10^−7^	873	30.9	[149]
GdBa_0.5_Sr_0.5_Co_2_O_5+δ_	4·10^−8^	923	47.2	[152]
GdBa_0.5_Sr_0.5_Co_1.5_Fe_0.5_O_5+δ_	3.0·10^−8^	873	34.7	[149]
GdBa_0.5_Sr_0.5_Co_1.5_Fe_0.5_O_5+δ_	5.13·10^−8^	923	40.6	[152]
GdBa_0.5_Sr_0.5_CoFeO_5+δ_	7.5·10^−8^	923	44.9	[149]
NdBa_0.5_Sr_0.5_Co_2_O_5+δ_	4·10^−8^	873	28.7	[152]
NdBa_0.5_Sr_0.5_Co_1.5_Fe_0.5_O_5+δ_	5.16·10^−8^	873	48.0	[149]
NdBa_0.5_Sr_0.5_CoFeO_5+δ_	3.8·10^−8^	873	28.6	[152]

**Table 7:** Performance (ASR: area specific resistance, MPD: maximal power density) of SOFCs based on *Ln*BaMe’Me”O_5+δ_ compounds.

Cathode	Electrolyte	ASR, Ω cm^2^ (*T*, K)	MPD, mW cm^–2^ (*T*, K)	Refs.
LaBaCuFeO_5+δ_	Ce_0.8_Sm_0.2_O_1.9_	0.21 (973)	557 (1073)	[176]
LaBaCuCoO_5+δ_	Ce_0.8_Sm_0.2_O_1.9_	0.11 (973)	603 (1073)	[176]
GdBaCuCoO_5+δ_	La_0.9_Sr_0.1_Ga_0.8_Mg_0.2_O_3–δ_	0.091 (1023)	545 (1073)	[177]
GdBaCuCoO_5+δ_	Sm_0.2_Ce_0.8_O_1.9_	0.129 (1023)	528 (1073)	[177]
SmBaCuCoO_5+δ_	BaCe_0.7_Zr_0.1_Y_0.2_O_3–δ_	0.22 (973)	355 (973)	[178]
PrBaCuCoO_5+δ_	Sm_0.2_Ce_0.8_O_1.9_	0.047 (973)	791 (973)	[179]
LaBaCuFeO_5+δ_	BaZr_0.1_Ce_0.7_Y_0.2_O_3–δ_	0.27 (973)	327 (973)	[181]
LaBaCuCoO_5+δ_	BaZr_0.1_Ce_0.7_Y_0.2_O_3–δ_	0.15 (973)	432 (973)	[181]
GdBaFeNiO_5+δ_	BaZr_0.1_Ce_0.7_Y_0.2_O_3–δ_	0.15 (973)	456 (973)	[183]
PrBaCoFeO_5+δ_	La_0.9_Sr_0.1_Ga_0.8_Mg_0.2_O_3–δ_	0.049 (1073)	749 (1073)	[184]
NdBaCoFeO_5+δ_	La_0.9_Sr_0.1_Ga_0.8_Mg_0.2_O_3–δ_	0.062 (1073)	669 (1073)	[184]
La_1.4_Ca_0.6_CoMnO_5+δ_	La_0.9_Sr_0.1_Ga_0.8_Mg_0.2_O_3–δ_	–	445 (1073)	[186]
NdBa_0.7_Ca_0.3_CoCuO_5+δ_	Gd_0.1_Ce_0.9_O_1.95_	0.038 (973)	1840 (1073)	[187]
Y_0.93_BaCoCuO_5+δ_	La_0.9_Sr_0.1_Ga_0.8_Mg_0.2_O_3–δ_	0.029 (1073)	862 (1123)	[188]
Pr_0.9_Ca_0.1_BaCoFeO_5+δ_	La_0.9_Sr_0.1_Ga_0.8_Mg_0.2_O_3–δ_	0.027 (1073)	728 (1073)	[190]

## Data Availability

Not applicable.
